# Exploring factors influencing the selection of primary health care delivery models in conflict-affected settings of North West and South West regions of Cameroon and North-East Nigeria: A study protocol

**DOI:** 10.1371/journal.pone.0284957

**Published:** 2023-05-03

**Authors:** Lundi-Anne Omam, Kelli O’Laughlin, Nicholas Tendongfor, Zara Wudiri, Mohammed Ngubdo Hassan, Alain Metuge, Ooju Oluwafemi, Esther Omam, Rosalind Parkes-Ratanshi

**Affiliations:** 1 Department of Public Health and Primary Care, University of Cambridge, Cambridgeshire, Cambridge, United Kingdom; 2 Department of Psychiatry, Clinical School, University of Cambridge, Cambridge, United Kingdom; 3 Departments of Emergency Medicine and Global Health, University of Washington, Seattle, WA, United States of America; 4 Department of Community Medicine, University of Maiduguri, Maiduguri, Nigeria; 5 Herwa Community Development Initiative, Maiduguri, Nigeria; 6 Reach Out Cameroon, Buea, Cameroon; 7 World Health Organisation, Abuja, Nigeria; 8 Infectious Diseases Institute, Makerere University College of Health Sciences, Kampala, Uganda; 9 Department of Public Health and Hygiene, University of Buea, Buea, Cameroon; University of Waterloo, CANADA

## Abstract

**Background:**

In conflict-affected settings, access to health care for displaced populations is constrained by barriers including geographical, cultural, communication, logistical, financial and insecurity. A six year humanitarian crises in the North West and South West regions of Cameroon has caused 27% of health facilities to be non-functional. The eleven year crisis in North-East Nigeria, has caused the closure of 26% of health facilities. These closure of health facilities and population displacement led to health care delivery using humanitarian funding by multiple different agencies. However, there is a paucity of evidence on the selection and design of the primary health care delivery models used in humanitarian settings. To ensure efficient use of resources and quality of services, model of care selection should be evidence based and informed by the specific humanitarian context. This research protocol aims to explore how primary health care models are selected by humanitarian organizations.

**Methods:**

We will conduct a cross sectional quantitative survey to map the range of primary health care delivery models used by humanitarian organisations in Cameroon and Nigeria. Using in-depth interviews and focus group discussions with staff from humanitarian organizations and internally displaced persons, we will explore the factors influencing the selection of primary health care models in these settings and determine the coverage and gaps in services across the different primary health care models. Quantitative data will be analysed in a descriptive manner and qualitative data will be analysed thematically.

**Discussion:**

Different models of care have been reported to be used by humanitarian organisations in conflict-affected settings, yet evidence on how different models are selected is lacking. A detailed understanding of the rationale for selection, the design and quality considerations of the strategies used to deliver health care will be obtained using a survey, in-depth interviews and focus group discussions.

## Background

More than one billion people currently live in countries with protracted crises caused by violence, conflicts or disasters. More so, populations living in conflict-affected regions face barriers to health care including geographic, cultural, communication, logistical, financial and security barriers [1–4]. Current estimates show that 53% of deaths in children under 5 years, 45% of neonatal deaths, and 60% of preventable maternal deaths occur in fragile, violent or conflict settings (commonly termed humanitarian settings) [5]. Significant morbidity and mortality in these populations could be mitigated with strong primary health care-oriented systems [3]. In most cases, humanitarian organisations and government institutions provide primary health care to the conflict-affected communities using various models of care [3]. However, evidence around choice and quality of these delivery models in humanitarian settings remain insufficient [6, 7].

Researchers have identified the need to conduct rigorous research on primary health care (PHC) models of care to improve access to essential health care [8–10]. But limited guidance on how to select models of care weakens programming efforts and undermines quality [8, 11]. Further, communities have an important role to play in designing and implementing humanitarian responses for sustainability [8, 11–13], yet they are seldom consulted during program design. So, it is unclear what truly determines what model of care to use under what circumstances in conflict settings.

Moreso, humanitarian organizations are often pressured by the humanitarian imperative to rapidly provide life-saving interventions and by donors to make use of limited resources in responses, yet evaluation of the quality of care is rarely undertaken. For example mobile clinics are reported to be expensive and logistically difficult to implement [8, 14], yet they are commonly used to provide primary health care in conflict-settings. Also, the Global Health Cluster Quality of Care in Humanitarian Settings guidelines and WHO’s quality of care in fragile, conflict-affected publication include 07 domains of quality (people-centered, safe, equitable, effective, timely, integrated and efficient) [5, 15, 16]. However, these do not include metrics on how to select PHC models or provide a guide for evaluation for quality of services. Furthermore, there is little underpinning primary research and published evidence to inform these guidelines. Therefore, there is a need to add scientific evidence to guideline development.

In the North West and South West (NWSW) regions of Cameroon, since 2017, an armed crises has caused the displacement of over 679,000 persons and destruction of 37% (253/933) health facilities [17]. In North East (NE) Nigeria, one of the most severely protracted crises in the world, has continued for over 11 years with 7.9 million people requiring humanitarian assistance and 1.8 million persons displaced [18]. The humanitarian crisis in North East Nigeria has led to the closure of 26% of health facilities. This destruction of health facilities and mass displacement of populations in both countries, has severely curtailed access to health care for these populations. This has resulted in an influx of humanitarian health organizations in both countries, providing essential health care through various models of care at the primary health care level [18–20].

Evidence on what guides and determines the model of care selection under different circumstances in conflict settings is limited. Understanding current models of care and the premise behind the selection of the model, is the first step in improving primary healthcare access and quality of care delivery in conflict settings. To address the evidence gap, this research will explore how primary health care delivery models are selected by humanitarian organizations. To provide baseline information which informed this protocol, a systematic review and mapping desk review were conducted from December 2020 to June 2021. The systematic review was conducted to document the different primary health care models used in conflict-affected settings in Africa, which has been published in a separate manuscript (reference to follow). The systematic review looked at detail information on the models of care used including location of delivery, human resources used, implementing institutions, services offered, cost, accessibility, and challenges. The results showed that the models of care in place in these conflict-affected settings include health facility-based, community-based interventions, mobile clinics, outreach and home visits as shown in Table 1. Primary health care for internally displaced persons and refugees is provided by a wide range of actors including: international and national non-governmental organisations, governments, United Nations agencies, community-based organisations, faith-based organisations and academic institutions.

**Table 1:** Models of care definitions derived from systematic review study.

Model of care	Definition	Alternative names	Personnel used
Health facility based	*Services offered at a fixed facility*.	Health centres, Clinics	Health care workers, Community health works (CHWs)
Community-based interventions	*Services are offered in the community*.	Community-based interventions, Community health workers, village health teams,	CHWs
		community health volunteers, lady health workers and community based distributors;	
		Traditional birth attendants,	
Outreach	*Services are offered as an advanced fixed strategy ran by at least 02 health personnel attached to a health facility or organisation. The teams do not rotate or move from one community to another*	Community outreach, fixed outreach site, aid post	Health Care Workers, CHWs
Mobile clinic	*A*mbulatory *services are offered at the community-based on a rotating basis*.	Mobile teams, mobile health center, mobile health clinic, health camps,	Health care workers
Home-visit	*Services are offered in the homes of the patients/beneficiaries*	Households visits, house-to-house visits, tent-to-tent visits	Health Care Workers, CHWs

In this protocol paper, we provide preliminary data from the mapping review on the primary health care landscape in NWSW Cameroon and NE Nigeria which was conducted in January 2021. This mapping review involved a selection of technical documents including situational reports and bulletins. To obtain data on humanitarian organizations in NWSW Cameroon and NE Nigeria, the Relief Web database [21] and the MSF database [22] were used to search for Humanitarian Situation Reports (SiTREPS) and health cluster (sector) bulletins; both of these are key documents during humanitarian responses. Additional documents or data were obtained by asking key informants about potentially useful technical documents available for public use. Publications were selected by titles solely for full reading and data extraction. A total of 24 publications were explored and entered on an excel spreadsheet. Data collected for this preliminary mapping included the title of the publication, source of data, date of publication, name of organization, type of organizations, primary health care services offered, what model of care was used for the last two years (2018 to 2020) and the location of services (per district or Division). Further information about this mapping process is included in S1 and S2 Appendices.

Data obtained from the preliminary study enabled the development of the research protocol presented in this paper. The aim of this paper is to present the scope, and methods that will be used to understand what drives the choice of a specific primary health care model of care, and what are the quality gaps for chosen models of care in NWSW Cameroon and NE Nigeria. The specific objectives in this study are 1) to map the different primary health care models used by humanitarian organisations in conflict-affected settings of NWSW Cameroon and NE Nigeria; 2) to explore the factors influencing the selection of primary health care models in this settings; and 3) to determine the coverage and gaps in services.

## Methods

### Study aim

This research protocol aims to explore how primary health care models are selected by humanitarian organizations.

### Study design

**Fig 1:**
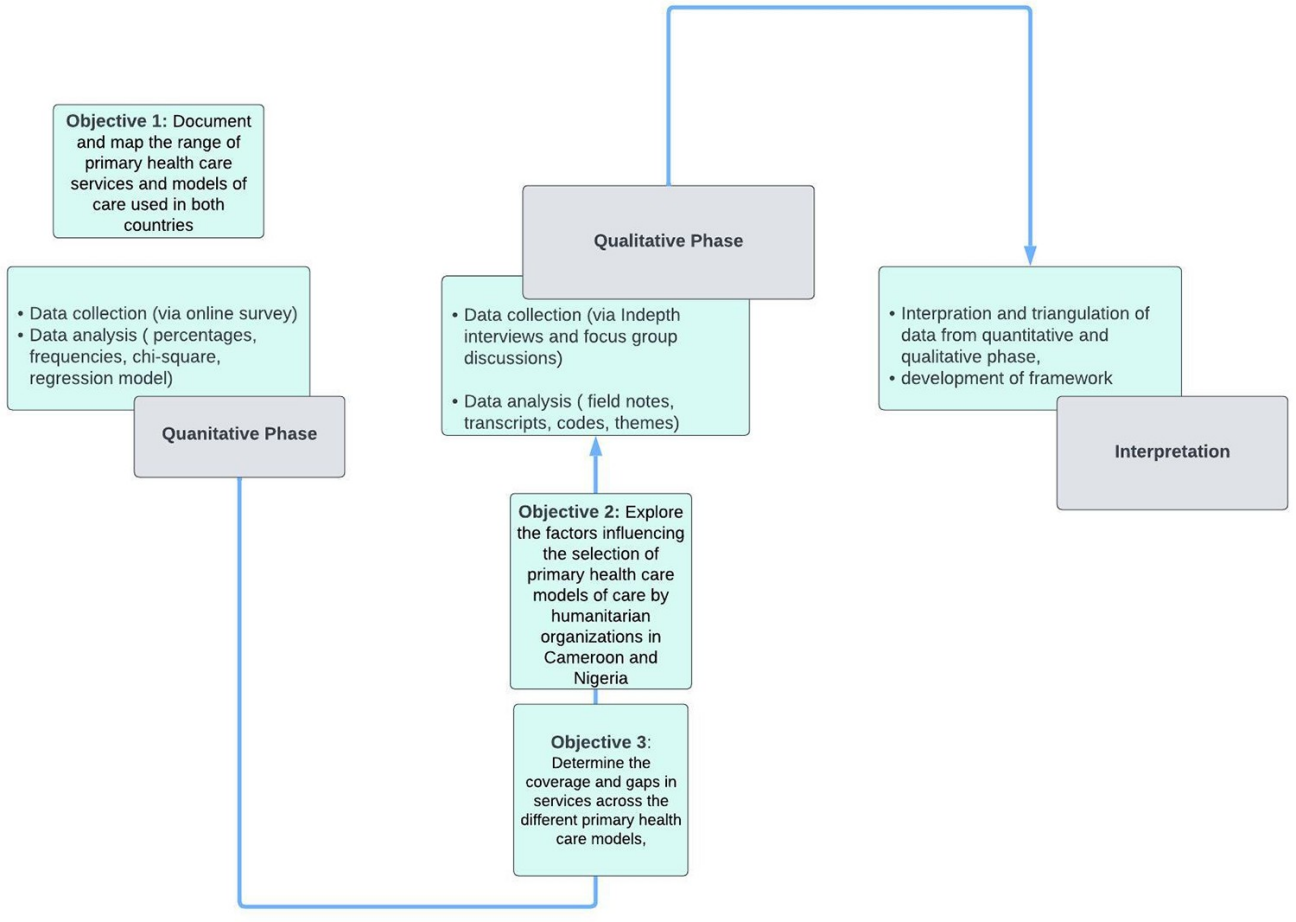
Flow chart of the research.

In order to evaluate the complex and multifaceted issues present in primary health care delivery to conflict-affected communities, this research will use a sequential explanatory mixed methods designs which begins with a quantitative phase using a survey followed by a qualitative phase including focus groups and in depth interviews [23–26]. The research project will take place over 18 months and will occur in the following phases:

*Quantitative Phase 1*: *Understanding the primary health care model of care landscape (months 1 to 5)*: To document and map the range of primary health care services and models of care used in both countries, a cross sectional survey will be conducted with all humanitarian organizations in the health clusters (clusters are groups of humanitarian organizations, both United Nations and non-United Nations, in each of the main sectors of humanitarian action) in the study areas who are willing to respond (Fig 1). The cross sectional survey will target project managers and senior staff in humanitarian organizations. A purposive sampling method will be used to recruit all organisations willing to participate. A list of humanitarian organisations will be obtained from the humanitarian sector or clusters. From this list, an email invite with a link to the online survey will be sent to all the organisations inviting them to participate in the study. Face-to-face meetings will also be used to recruit and invite participants to take the online survey. One questionnaire will be administered per organization. Examples of survey variables will include organizational characteristics, models of care used, services offered, location of services, beneficiary populations (socio-demographics, ethnicity, gender, types of displacement).

*Qualitative Phase 2a*: *Determining model of care selection factors (months 5 to 10)*: To explore the factors influencing the selection of primary health care models of care by humanitarian organizations in Cameroon and Nigeria (Table 2, mapping review results demonstrating existing models of care in both settings), a qualitative study using focus group discussions and in-depth interviews will be conducted (Fig 1). Study participants will include staff from humanitarian organization, ministry of health staff and internally displaced persons. Study participants from humanitarian organisations will be purposefully targeted and recruited using a screening tool with specific inclusion and exclusion criteria to identify the type of participants to be recruited for the in-depth interviews. A range of age, seniority, gender, roles, geography and types of primary health care models experienced will be enrolled through purposeful maximum variation sampling. Focus group discussions will be undertaken with internally displaced persons, identified through community facilitators used by humanitarian organizations. For the Focus group discussions, a local level mapping process will be conducted with key informants to determine the details of internally displaced persons residing within the district or local government area. A screening tool will be used to screen and identify internally displaced persons of different age, gender, ethnicity and/or socio-economic status. The in-depth interviews will be mapped according to the number and details of available groups of in-depth interviews at the districts/local government area, origin and occupation. Targeted sampling will be used to recruit participants for the in-depth interviews and focus group discussions. An minimum total of 30 in-depth interviews and 10 focus group discussions will be conducted in both countries. Interviews will be conducted until saturation is achieved. Themes to be explored during interviews with humanitarian organisations and ministry of health staff include location of interventions, security and access, community engagement, health facilities’ functionality, acceptability of models, barriers to accessing care, displacement patterns, ministry of health priorities, and funder priorities, barriers to health care.

**Table 2:** Mapping review results demonstrating models of primary health care and services offered in Cameroon and Nigeria.

Models of PHC care	Services offered	Cameroon (N)	Nigeria (N)
Community based interventions	Community based COVID-19 response, community management of HIV&TB, Essential health services, Early warning and alert system (EWARS), Health promotion, reproductive health	7	17
Mobile clinics	Essential health services, EWARS, immunization, mental health, reproductive health	17	35
Outreach	Immunization, mental health, reproductive health	0	17
Health Facility Based	Emergency care, Essential health services, immunization, reproductive health	1	44
Mobile Clinics & Health facility based	Essential Health services, reproductive health,	1	33
Government services	Immunization	9	0

For internally displaced persons, themes to be explored include; different strategies (models of care) available, models of care accessibility, community participation in model of care design and selection.

*Qualitative phase 2b*: *Determining the quality of care gaps (months 10–15)*: To determine the coverage and gaps in services across the different primary health care models, we will undertake further in-depth interviews with front line humanitarian program staff, ministry of health district staff and internally displaced persons [5, 27]. For the in-depth interviews, purposeful sampling will also be used to enroll participants of different ages, sex, ethnicity and displacement status. A minimum of 10 in-depth interviews will be conducted; interviews will be conducted until saturation is achieved. Themes to be explored will include gaps in service provision and quality of care (focusing on the quality of care domains: patient centered, safe, equitable, effective, integrated, timely and efficient). Same themes will be explored for both the different groups of study participants. However, the questions for IDPs will be simplified.

*Interpretation Phase*: *Triangulation of data from quantitative and qualitative phase*, *and developing the model of care framework (months 15–18)*: This final phase of the research will involve integrating results from both the quantitative phase and qualitative phase to inform the development of the framework to guide the selection of primary health care models and a “quality toolkit” for monitoring a pragmatic set of quality interventions for the different models of care in conflict settings (Fig 1). The framework will be developed during consultative workshops with key stakeholders from humanitarian organizations, government, and communities. This workshop will be organized to first share results of the studies conducted (for contextual validation) and to collectively design a quality toolkit for monitoring service delivery with different models of care. A total of two workshops with at least 30 participants each will be conducted.

### Study setting

**Fig 2:**
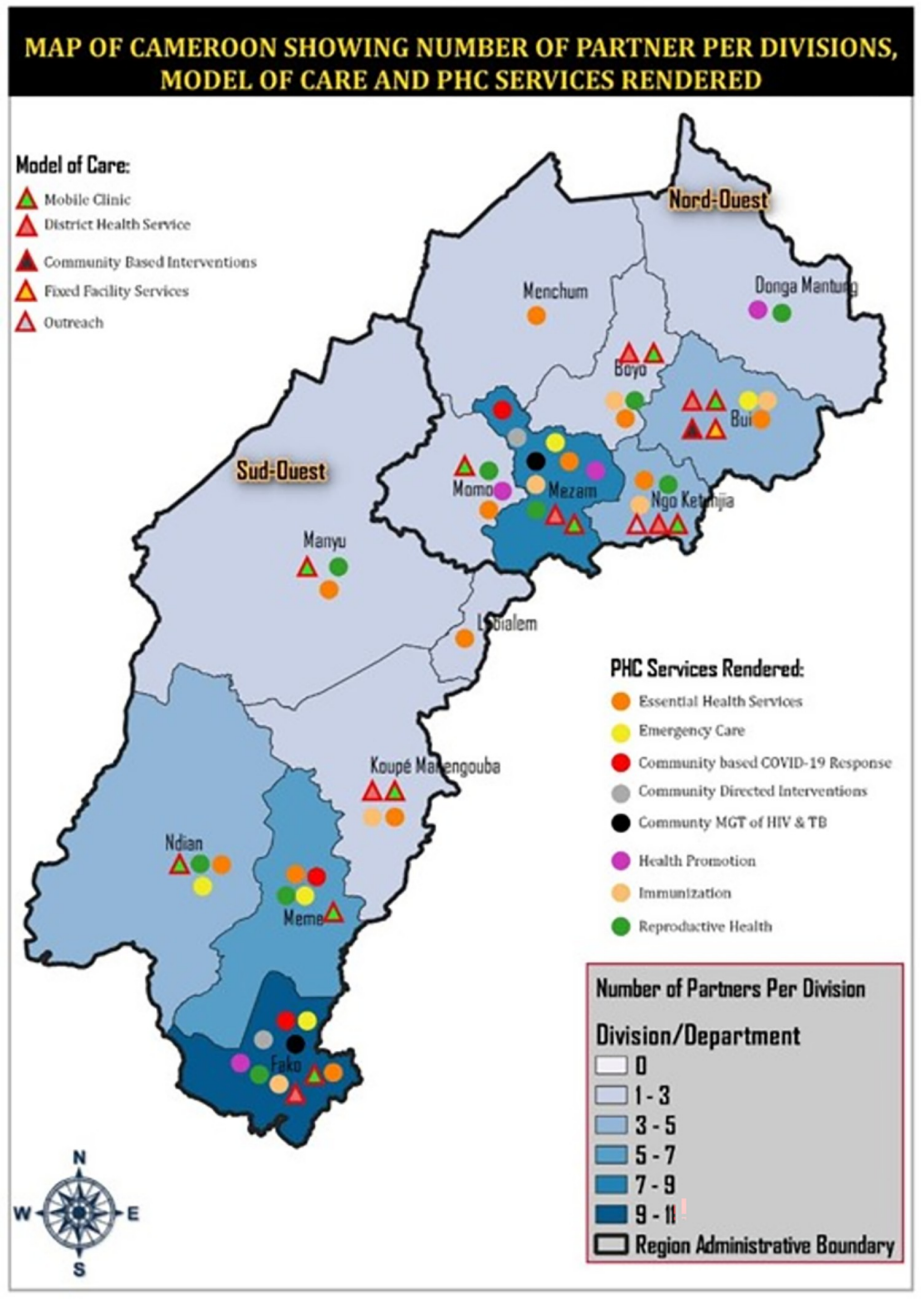
Mapping review results demonstrating primary health care models of care and services in the North West and South West regions of Cameroon. Republished from [httpsgrid3.org) and https://data.humdata.org)] under a CC BY license, with permission from Ooju Oluwafemi.

**Fig 3:**
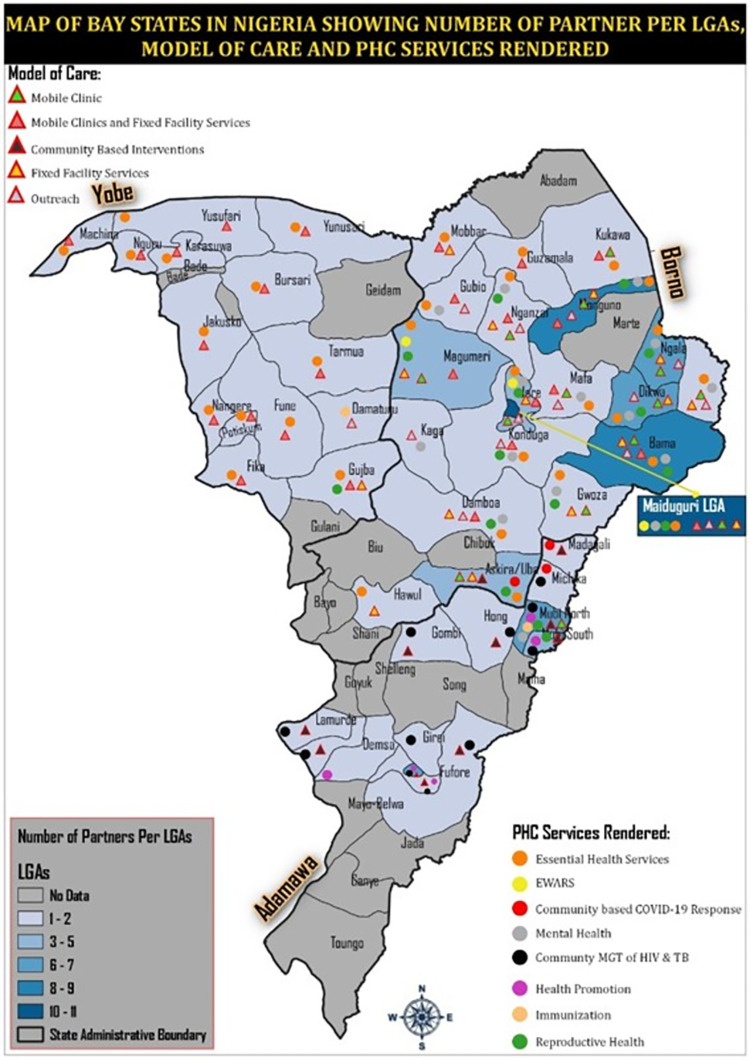
Mapping review findings demonstrating primary health care models of care and services in North East Nigeria. Republished from [https://grid3.org) and https://data.humdata.org)] under a CC BY license, with permission from Ooju Oluwafemi.

The research will be conducted in the NWSW Cameroon and NE Nigeria, two countries with protracted conflict crises. In Cameroon, communities with most operations of humanitarian organizations as revealed by the mapping review (Fig 2) will be included in the study. In Northeast Nigeria, communities from Borno, Adamawa and Yobo will be included in the research. The majority will be in Borno State, which is the epicenter of the crises as revealed from the results of the mapping review (Fig 3).

### Study population

The research will be undertaken with adult staff of humanitarian organizations and internally displaced populations. To ensure the research is gender sensitive and inclusive, the study participants will be purposefully recruited to include the voices and inputs of men and women of diverse age groups, ethnicity, religion and race. Project managers, project supervisors, grant writers and frontline workers of humanitarian organizations (e.g., United Nations agencies, international non-governmental organizations, national non-governmental organizations and community-based organizations) will be interviewed in this research. Interviews will be conducted as well with internally displaced persons in urban, peri-urban, rural and hard-to-reach areas. We will purposefully select participants from across different geographical area to ensure wide coverage of experiences/views. Regions and states as well as implementing partners in these areas will be grouped into urban, peri-urban and rural.

### Inclusion criteria

**Phase 1.** For the first phase of the research, male and female adult staff of humanitarian organisations and Ministry of Health representatives who are 21 year or older and are able to provide informed consent will be included in the study.

**Phase 2a and 2b.** In the second phase of the research, male and female adult staff of humanitarian organisations and Ministry of Health staff who are 21 year or older and are able to provide informed consent will be included in the study. Also, internally displaced persons (confirmed to be a resident of an IDP or host community) who are 21 years of age or older and are able to provide informed consent will be included in the second to the fourth phase of the research.

**Phase 3.** In this phase of the research, male and female adult staff of humanitarian organisations and Ministry of Health representatives who are 21 year or older and are able to provide informed consent will be included in the study. Also, internally displaced persons (confirmed to be a resident of an IDP or host community) who are 21 years of age or older and are able to provide informed consent will be included in this phase of the research.

### Exclusion criteria

**Phases 1–3.** Throughout the study, individuals who are mentally ill or have limitations to communicate well or cannot speak or understand the local languages will be excluded from the study. Also, those who refuse to provide consent to the study or who do not want to participate in the study.

### Recruitment (selection) of study participants

For the cross sectional survey, a link with the already uploaded questionnaire on Koko Collect will be sent by email to all humanitarian organisations in OCHA’s data base. The link will also be sent to Ministry of Health representatives. One questionnaire will be administered per organization and program managers from each organization will be solicited as interviewees.

For phase 2a and 2b of the research, with humanitarian organisations, participants will be enrolled by targeted sampling from identified humanitarian organizations. A screening tool (S3 Appendix) with specific inclusion and exclusion criteria will be develop to identify the type of people to be recruited for the interviews. To recruit participants for the focus group discussions and in-depth interviews, a local level mapping process will be conducted with community key informants to determine the details of IDPs residing within the district or local government area. The IDPs will be mapped according to the number and details of available groups of IDPs at the districts/LGA, origin and occupation. Within each community, the required number of IDPs will be selected purposively such that it reflects different age groups and gender. When there is less information available from the key informants, snowballing will be used to detect harder-to-reach IDPs.

### Sample size

**Phase 1.** An estimated sample size of 100 humanitarian organisations in both Cameroon and Nigeria will be included in the study. These sample sizes were estimated based on results obtained from the mapping review (Table 3). In Cameroon, we anticipate sampling a minimum of 20 organizations in South West region and 20 organizations in the North West region. In North East Nigeria we anticipate sampling a minimum of 60 organizations, 40 in Borno, 10 in Yobe and 10 in Adamawa.

**Table 3:** Summary of important features from the mapping review.

Dimension	Categories (N)		Total Reported (N)
Location	NWSW Cameroon (34)	NE Nigeria (40)	74 organisations
Region or States	SW (20), NW (14)	Yobe (7), Borno (17), Adamawa (16)	74 organisations,
Provider	UN agency (15), INGO (25), NNGO (27), FBO (2), MoH (2)		74 providers
PHC services	Community Directed Interventions (2), Community management of HIV&TB (17), Community based COVID-19 response (15), Essential Health Services (107), Emergency care (6), EWARS (6), Health Promotion (19), Reproductive Health (46), Mental Health (24), Immunization (13)		254 locations where services were offered 10 PHC services

NWSW = North West and South West; NEN = North East Nigeria; NW = North west; SW = South West; UN = United Nations; INGO = International non-governmental organisations; FBO = Faith based organisations; MoH = Ministry of Health; NNGO = national non-governmental organisations; PHC = primary health care; EWARS = early warning and alert system.

**Phase 2a and 2b.** Estimated sample size for the in-depth interviews is 40 (18 IDIs in Cameroon and 22 IDIs in Nigeria). For the focus group discussion, estimated sample size is 10 (S4 Appendix). For both in-depth interviews and focus group discussions, interviews will be conducted until saturation is attained.

**Phase 3.** Estimated number of participants for the stakeholders workshop are 30 for Cameroon and 30 for Nigeria.

### Data collection

Quantitative data from the cross sectional survey will be collected through a structured questionnaire. Gender sensitive languages will be used in our research questionnaires and interview guides. The survey will be hosted online using the Kobo Collect toolbox and a link to the survey sent by email to all humanitarian organizations and Ministry of Health. For organizations that will not be reached by email, face-to-face interviews will be undertaken.

Qualitative data will be collected through in-depth interviews and focus group discussions. The in-depth interviews will be led by a trained interviewer and a research assistant for notes taking. The location and time of the interview will be agreed upon by both the interviewer and interviewee to allow for privacy. Due to security or COVID-19 concerns, some interviews will also be administered online by teleconference. This will be agreed upon by the interviewer and the interviewee. Interviews will run from 30 to 60 minutes. The interviewer and research assistant will be provided with basic personal protective equipment like facemasks, hand sanitisers and appropriate physical distancing will be observed, in line with national guidance. For online interviews, the computer, internet credit and recording device will be provided by the researcher during interviews. Interviews will be conducted in the English, pidgin English, Hausa and any other local language, with notes being written down complemented by audio-recording. Trained translators will be used during interviews conducted with internally displaced persons. All interviews will be transcribed in the languages in which they were conducted before being translated into English.

To ensure high quality data, the interview guides will be pre-tested prior to the start of data collection. Quality of data will also be ensured through a comprehensive feedback and supervision process. In addition, all interviewers will receive intensive training on data collection prior to commencing data collection activities. The focus group discussions will be led by a trained facilitator and a research assistant.

### Data analysis

#### Quantitative data

Once entered, the data will be cleaned, merged, and then exported to R for analysis. Data will be analyzed in a descriptive manner. Logistic regression models will be used to view possible associations while avoiding confounding effects [27].

#### Qualitative data

Qualitative Data from focus group discussions and in-depth interviews will be recorded on tape recorders, transcribed and translated into English. In-depth Interviews will be analyzed thematically using coding to identify emergent patterns, concepts and categories from participants’ accounts. Initially, open, descriptive coding will be used to ensure interpretations are grounded in participants’ accounts as much as possible, and that findings are generated inductively, as well as explore pre-determined areas of investigation. The analysis will be conducted using NVivo® software.

All data analyzed in this research would be disaggregated by sex and age. Policy recommendations from this research would be described such that it presents equal opportunities for men, women, persons living with disabilities, and ethnic minorities.

### Ethics approval and consent to participate

This research partnership includes three academic institutions and two operational humanitarian organizations. Preparations for the study took place from October 2020 to May 2021. The partners were brought together by the University of Cambridge researchers and considerations were given to invite two humanitarian organizations and two academic institutions in Cameroon and Nigeria to provide support with contextual research experience and humanitarian access/mediation. A memorandum of understanding was established and signed by all partners in the consortium to ensure commitment from all partners.

Ethics approval has been obtained from the University of Buea faculty of health science–Institutional review board (*2021/1513-07/UB/SG/IRB/FHS*), the National ethics committee for research in human health Cameroon (N°2022/02/1435/CE/CNERSH/SP), the National Health Research Ethics Committee of Nigeria (*NHREC/01/01/2007-27/09/2021*) and the Cambridge Psychology Research Ethics Committee *(PRE*.*2021*.*106)*. Administrative approval for this research has been obtained from the South West and North West Regional Delegations of Public Health, Ministry of Health in Cameroon and the Commissioner for Health for Borno/Adamawa, Yobe, Ministry of Health Nigeria. Verbal and written consent will be obtained from all participants of this study.

### Confidentiality and data protection

During collection of primary data, participant’s confidentiality will be ensured by removing any personal information from completed survey and interview instruments. Access to master key codes will be limited and master lists will be stored separately from the data and destroyed as soon as reasonably possible. Contact lists and recruitment records will be destroyed when no longer required for the research. Files containing electronic data will be password-protected and encrypted. Files containing electronic data will be closed when computers will be left unattended. Consent forms will be stored securely in locked cabinets or rooms, separately from the research data. Finally, research assistants will be trained in the IRB-approved methods for managing and storing research data.

### Anticipated challenges and risks

There are some challenges and anticipated risk associated with conducting rigorous studies in conflict-affected settings. First, due to the high levels of insecurity from the active presence of state and non-state armed groups in both countries, there are high risks of kidnapping, crossfire, and forced retention of the research team members. However, we will work closely with local non-governmental organizations who have experience working in these areas for many years, with security protocols enforced strictly. Also, constant negotiation for access and participation in local humanitarian access working groups will be done to ensure the safety of research team members. Utilizing local staff in rural areas will increase acceptance and functionality of the research. Additionally, most data collection and trainings will be moved to online and/or safe sites when study sites become inaccessible.

The second challenge is linked to the risk of psychological trauma to internally displaced persons who might recall traumatic experiences they have been facing during the conflict. The research team during recruitment of participants will provide the information sheet to all research participants to read about the research or will be read aloud to them if they are not literate so they could decide whether to be part of the research or not. Also, the interviewers will be trained to stop the interview if it is observed that the interview process is bringing memories that are affecting the research participants emotionally. Participants who show signs of distress during the interview will be linked to organizations providing mental health support for follow up.

Another anticipated risk of our research is that of interview fatigue to the participants. This is because most of the interviews will run for more than 30 minutes, there is a risk for research participants to loose time that would have been used otherwise to their benefit. To mitigate this risk, the research team will inform the research participants about the time that will be required to take part in the research. This prior information will enable the research participants to plan their other activities to minimize waste of time. The research will also compensate research participants for their time with internet or transport vouchers.

Further, an anticipated risk is that of coercion and undue influence to study participants especially with internally displaced person who might feel pressured to take part in the study. To mitigate this risk, we will take time to explain to the study participants that they are free to not take part in the study and are free not to respond to questions they do not want to. This is explained in the information letter and consent form as well. To avoid coercion- we will also select compensation that will be reasonable but not likely to entice them to participate in the research.

Furthermore, because our research will be conducted in the context of the COVID-19 pandemic which might pose a risk of infection to the research team and study participants, we envisage respecting COVID-19 national protocols and standard operating procedure with infection prevention and control measures. Teleconference interviews will be used in the research in preference to face-to-face interviews.

## Discussion

This study is of great importance as it is being undertaken in an understudied context of humanitarian crises and it has the potential to inform the practice of selecting effective primary health care models of care by humanitarian actors in conflict-affected areas. The use of mixed methods in the proposed research is ideal for understanding the primary health care landscape and understanding what drives the use of model of care selection in these conflict affected settings [28–30].

Currently multiple different models of care have been reported as used by humanitarian organisations in conflict-affected settings [8, 10, 31–33], yet evidence on how different models are selected is lacking. In our desktop review we identified 74 organisation in Nigeria and Cameroon that were providing primary care at 254 sites in conflict affected areas. We found that mobile clinics, community-based interventions, and health facility based models of care are the most common approaches of delivering health care to communities. We have used the mapping survey to develop this research protocol which determines the collection of new data in order to ascertain the reason behind the choice of these models of care. Furthermore, it is recommended that humanitarian health interventions should mostly target areas with partially or non-functional health facilities as a system strengthening approach [15]. This prevents duplication of existing services or undermining the local health system. However, it is currently not clear from existing literature or our desktop mapping if humanitarian operations were mostly in communities with weakened health systems or not. A rigorously designed quantitative survey will give a better picture of models of care currently used in these settings. A detailed understanding of the rationale for selection, the design and quality considerations of the strategies used to delivery health care will be obtained by deeper interrogation of the issues using in-depth interviews and focus group discussions.

One of the main outcomes of this research will be the development of a framework to guide the selection of primary health care models and a “quality toolkit” for monitoring a pragmatic set of quality interventions for the different models of care in conflict settings. For this to be achieved, consultative/advocacy workshops with key stakeholders from humanitarian organizations, government and communities will be organized to share the results and collectively draft a guide and quality toolkit for monitoring services delivery with different models of care. It is hoped that the research outcomes will impact and inform humanitarian health programming policies in Africa. The impact of the primary health care model of care framework and toolkit on quality of care by humanitarian organizations could be evaluated in future research using randomized controlled trials.

### Strengths and limitations

To our knowledge, our study will be the first to explore how primary health care delivery models are selected by humanitarian organizations in conflict settings of Cameroon and Nigeria. Combining quantitative and qualitative methods in exploring the drivers behind the choice and use of different models of care provides breadth and depth to this research. Our research will lead to the development of a framework aimed at guiding the selection of models of care in humanitarian settings in low- and middle-income countries. To our knowledge no such guide exist and this research will provide a foundation on which humanitarian organisations can refer for programming orientation. The mapping review conducted to inform the primary data collection phase enabled us to design this study to suit context realities, considering the fragile and complex nature of the armed conflict in the study areas.

A limitation associated with this research is the security risks due to the active conflict in both countries. Furthermore, the COVID-19 pandemic adds challenges related to minimizing face-to-face contact. Some of the interviews will be online which limits thorough observation of body languages which can be informative in qualitative studies.

## Conclusion

To take the next step in ensuring accessible and quality primary health care is delivered in humanitarian crises, it is first important that we understand current models of care and reasons for their selection. In the proposed research, we will map the different primary health care delivery models used in conflict settings of Cameroon and Nigeria, explore the factors influencing the selection of these models of care, determine the coverage and gaps in services across the different primary health care models and develop a framework to guide the selection of these models of care.

To our knowledge, this study will be the first to understand the primary health care landscape in conflict settings including choice of PHC models and quality interventions. Without this information, designing and selecting models of care that are best suited under specific contexts and are of good quality is not possible.

## Supporting information

S1 Appendix Extracted Data from Desk review for mapping of PHC models of care and services in Cameroon and Nigeria. (DOCX)

S2 Appendix Methodology used in mapping the primary health care services. (DOCX)

S3 Appendix Screener for In-depth interviews and focus group discussions. (DOCX)

S4 Appendix Anticipated number of study participants by study phase and component. (DOCX)

## Abbreviations

CHWs: Community Health Workers
EWARS: Early warning and alert system
FBO: Faith based organisations
INGO: International non-governmental organisations
MoH: Ministry of Health
NEN: North East Nigeria
NNGO: National non-governmental organisations
NWSW: North west and South West Regions
NW: North west
PHC: Primary health care
SW: South West
UN: United Nations
