# Supplementary material for: Exploring factors influencing the selection of primary health care delivery models in conflict-affected settings of North West and South West regions of Cameroon and North-East Nigeria: A study protocol
Source: PLoS One. 2023 May 3;18(5):e0284957. doi: 10.1371/journal.pone.0284957 (PMC10155952; doi:10.1371/journal.pone.0284957)
Supplement: S1 Appendix — (DOCX) [file pone.0284957.s001.docx]

## **APPENDIX 1**

## **Extracted Data from Desk review for mapping of PHC models of care and services in Cameroon and Nigeria**

| **Name of publication** | **Publishing organisation** | **Date of publication** | ***Name of organisation or institution*** | ***Type of Organisation*** | ***Country of operation*** | ***Region or state of where PHC Services were offered*** | ***Division where PHC Services are rendered*** | ***District, Division or local government areas PHC Services are offered*** | ***PHC Services offered*** | ***Model ofCare used*** |
| --- | --- | --- | --- | --- | --- | --- | --- | --- | --- | --- |
| SITREP NWSW | OCHA | 30th April 2020 | CARITAS | Faith Based Organisation | Cameroon | NW | Boyo |  | Essential Health Services | Mobile Clinics |
| SITREP NWSW | OCHA | 31st July 2020 | RDPH | Ministry of Health | Cameroon | NW | Boyo | Fundong | Immunization | District Health Service |
| SITREP NWSW | OCHA | 30th June 2019 | Care International | INGO | Cameroon | NW | Boyo |  | Reproductive Health |  |
| SITREP NWSW | OCHA | 30th April 2020 | CARITAS | Faith Based Organisation | Cameroon | NW | Bui |  | Essential Health Services | Mobile Clinics |
| SITREP NWSW | OCHA | 31st July 2020 | CBCHS | Faith Based Organisation | Cameroon | NW | Bui |  | Immunization | Fixed Facility Services |
| SITREP NWSW | OCHA | 31st October 2019 | CBCHS | Faith Based Organisation | Cameroon | SW | Bui | Kombo | Essential Health Services | Community Based Interventions |
| NWSW Operational Presence | OCHA | Accessed 19/01/2021 | WHO | UN Agency | Cameroon | NWSW | Bui |  | Emergency Care |  |
| NWSW Operational Presence | OCHA | Accessed 19/01/2021 | H4BF | National NGO | Cameroon | NWSW | Donga-Mantung |  | Health promotion |  |
| NWSW Operational Presence | OCHA | Accessed 19/01/2021 | H4BF | National NGO | Cameroon | NW | Donga-Mantung |  | Reproductive Health |  |
| NWSW Health Cluster Bulletin | Health Cluster | 25th June 2020 | RDPH | Ministry of Health | Cameroon | SW | Fako | Buea | Immunization | District Health Service |
| NWSW Health Cluster Bulletin | Health Cluster | 25th June 2020 | RDPH | Ministry of Health | Cameroon | SW | Fako | Tiko | Immunization | District Health Service |
| NWSW Health Cluster Bulletin | Health Cluster | 25th June 2020 | RDPH | Ministry of Health | Cameroon | SW | Fako | Muyuka | Immunization | District Health Service |
| NWSW Health Cluster Bulletin | Health Cluster | 25th June 2020 | RDPH | Ministry of Health | Cameroon | SW | Fako | Limbe | Immunization | District Health Service |
| SITREP NWSW | OCHA | 30th April 2020 | Reach Out Cameroon | National NGO | Cameroon | SW | Fako | Muyuka | Essential Health Services | Mobile Clinics |
| NWSW Health Cluster Bulletin | Health Cluster | 13th May 2020 | Superior Health Foundation | National NGO | Cameroon | SW | Fako |  | Health promotion |  |
| NWSW Health Cluster Bulletin | Health Cluster | 16th July 2020 | Reach Out Cameroon | National NGO | Cameroon | SW | Fako |  | Community based COVID-19 response |  |
| NWSW Health Cluster Bulletin | Health Cluster | 23rd August 2020 | CUAPWD | National NGO | Cameroon | SW | Fako |  | Health promotion |  |
| SITREP NWSW | OCHA | 31st October 2019 | UNFPA | UN Agency | Cameroon | SW | Fako |  | Reproductive Health |  |
| SITREP NWSW | OCHA | 31st October 2019 | LUKMEF | National NGO | Cameroon | SW | Fako | Buea | Essential Health Services | Mobile Clinics |
| SITREP NWSW | OCHA | 31st October 2019 | Action Against Hunger | INGO | Cameroon | SW | Fako |  | Essential Health Services | Mobile Clinics |
| SITREP NWSW | OCHA | 30th June 2019 | Action Against Hunger | INGO | Cameroon | SW | Fako |  | Essential Health Services | Mobile Clinics |
| SITREP NWSW | OCHA | 30th June 2019 | Care International | INGO | Cameroon | SW | Fako |  | Reproductive Health |  |
| SITREP NWSW | OCHA | 31st July 2019 | Relief International | INGO | Cameroon | SW | Fako |  | Essential Health Services |  |
| NWSW Operational Presence | OCHA | Accessed 19/01/2021 | Reach Out Cameroon | National NGO | Cameroon | SW | Fako | Muyuka | Community Directed Interventions |  |
| NWSW Operational Presence | OCHA | Accessed 19/01/2021 | Reach Out Cameroon | National NGO | Cameroon | SW | Fako | Buea | Communty Management of HIV&TB |  |
| NWSW Operational Presence | OCHA | Accessed 19/01/2021 | Reach Out Cameroon | National NGO | Cameroon | SW | Fako | Buea | Community based COVID-19 response |  |
| NWSW Operational Presence | OCHA | Accessed 19/01/2021 | Reach Out Cameroon | National NGO | Cameroon | SW | Fako | Limbe | Community based COVID-19 response |  |
| NWSW Operational Presence | OCHA | Accessed 19/01/2021 | ECOCAM | National NGO | Cameroon | SW | Fako | Limbe | Community based COVID-19 response |  |
| NWSW Operational Presence | OCHA | Accessed 19/01/2021 | WHO | UN Agency | Cameroon | NWSW | Fako |  | Emergency Care |  |
| SITREP NWSW | OCHA | 31st July 2020 | RDPH | Ministry of Health | Cameroon | NW | Kombo East |  | Immunization | District Health Service |
| NWSW Health Cluster Bulletin | Health Cluster | 25th June 2020 | RDPH | Ministry of Health | Cameroon | SW | Kupe-Muanenguba | Tombel | Immunization | District Health Service |
| SITREP NWSW | OCHA | 30th April 2020 | Reach Out Cameroon | National NGO | Cameroon | SW | Kupe-Muanenguba |  | Essential Health Services | Mobile Clinics |
| SITREP NWSW | OCHA | 31st October 2020 | CARITIS | Faith Based Organisation | Cameroon | SW | Lebialem |  | Essential Health Services |  |
| SITREP NWSW | OCHA | 30th April 2020 | Reach Out Cameroon | National NGO | Cameroon | SW | Manyu |  | Essential Health Services | Mobile Clinics |
| SITREP NWSW | OCHA | 31st October 2019 | UNFPA | UN Agency | Cameroon | SW | Manyu |  | Reproductive Health |  |
| SITREP NWSW | OCHA | 31st October 2020 | CARITIS | Faith Based Organisation | Cameroon | SW | Manyu |  | Essential Health Services |  |
| NWSW Operational Presence | OCHA | Accessed 19/01/2021 | Reach Out Cameroon | National NGO | Cameroon | SW | Manyu | Eyumojock | Essential Health Services | Mobile Clinics |
| SITREP NWSW | OCHA | 30th April 2020 | CARITAS | Faith Based Organisation | Cameroon | SW | Meme |  | Essential Health Services | Mobile Clinics |
| SITREP NWSW | OCHA | 31st October 2019 | UNFPA | UN Agency | Cameroon | SW | Meme |  | Reproductive Health |  |
| SITREP NWSW | OCHA | 31st October 2019 | Action Against Hunger | INGO | Cameroon | SW | Meme |  | Essential Health Services | Mobile Clinics |
| SITREP NWSW | OCHA | 30th June 2019 | Care International | INGO | Cameroon | SW | Meme |  | Reproductive Health |  |
| SITREP NWSW | OCHA | 31st October 2020 | CARITIS | Faith Based Organisation | Cameroon | SW | Meme |  | Essential Health Services |  |
| NWSW Operational Presence | OCHA | Accessed 19/01/2021 | Reach Out Cameroon | National NGO | Cameroon | SW | Meme | Kumba | Community based COVID-19 response |  |
| NWSW Operational Presence | OCHA | Accessed 19/01/2021 | WHO | UN Agency | Cameroon | NWSW | Meme |  | Emergency Care |  |
| SITREP NWSW | OCHA | 31st October 2020 | CARITIS | Faith Based Organisation | Cameroon | NW | Menchum |  | Essential Health Services |  |
| SITREP NWSW | OCHA | 30th April 2020 | CARITAS | Faith Based Organisation | Cameroon | NW | Mezam |  | Essential Health Services | Mobile Clinics |
| SITREP NWSW | OCHA | 31st July 2020 | RDPH | Ministry of Health | Cameroon | NW | Mezam | Bamenda | Immunization | District Health Service |
| NWSW Health Cluster Bulletin | Health Cluster | 16th July 2020 | HEDECS | National NGO | Cameroon | NW | Mezam |  | Community based COVID-19 response |  |
| NWSW Health Cluster Bulletin | Health Cluster | 2rd July 2020 | Strategic Humanitarian Services (SHUMAS) | National NGO | Cameroon | NW | Mezam |  | Community based COVID-19 response |  |
| SITREP NWSW | OCHA | 31st October 2019 | UNFPA | UN Agency | Cameroon | SW | Mezam |  | Reproductive Health |  |
| SITREP NWSW | OCHA | 30th June 2019 | Care International | INGO | Cameroon | NW | Mezam |  | Reproductive Health |  |
| NWSW Operational Presence | OCHA | Accessed 19/01/2021 | HEDECS | National NGO | Cameroon | NW | Mezam | Bamenda | Community Directed Interventions |  |
| NWSW Operational Presence | OCHA | Accessed 19/01/2021 | COHESODEC | National NGO | Cameroon | NW | Mezam |  | Communty Management of HIV&TB |  |
| NWSW Operational Presence | OCHA | Accessed 19/01/2021 | WHO | UN Agency | Cameroon | NWSW | Mezam |  | Emergency Care |  |
| NWSW Operational Presence | OCHA | Accessed 19/01/2021 | HEDECS | National NGO | Cameroon | NW | Mezam |  | Health promotion |  |
| NWSW Operational Presence | OCHA | Accessed 19/01/2021 | H4BF | National NGO | Cameroon | NW | Mezam |  | Reproductive Health |  |
| SITREP NWSW | OCHA | 30th April 2020 | Reach Out Cameroon | National NGO | Cameroon | NW | Momo |  | Essential Health Services | Mobile Clinics |
| SITREP NWSW | OCHA | 30th June 2019 | Care International | INGO | Cameroon | NW | Momo |  | Reproductive Health |  |
| NWSW Operational Presence | OCHA | Accessed 19/01/2021 | ALIMA | INGO | Cameroon | NW | Momo | Batibo | Health promotion |  |
| SITREP NWSW | OCHA | 30th April 2020 | CARITAS | Faith Based Organisation | Cameroon | SW | Ndian |  | Essential Health Services | Mobile Clinics |
| SITREP NWSW | OCHA | 31st October 2019 | UNFPA | UN Agency | Cameroon | SW | Ndian |  | Reproductive Health |  |
| SITREP NWSW | OCHA | 31st October 2019 | Reach Out Cameroon | National NGO | Cameroon | SW | Ndian | Ekondo-Titi | Essential Health Services | Mobile Clinics |
| SITREP NWSW | OCHA | 30th June 2019 | Care International | INGO | Cameroon | SW | Ndian |  | Reproductive Health |  |
| NWSW Operational Presence | OCHA | Accessed 19/01/2021 | WHO | UN Agency | Cameroon | NWSW | Ndian |  | Emergency Care |  |
| SITREP NWSW | OCHA | 31st July 2020 | RDPH | Ministry of Health | Cameroon | NW | Ndop |  | Immunization | District Health Service |
| SITREP NWSW | OCHA | 30th April 2020 | Reach Out Cameroon | National NGO | Cameroon | NW | Ngoketunjia |  | Essential Health Services | Mobile Clinics |
| SITREP NWSW | OCHA | 31st July 2020 | CBCHS | Faith Based Organisation | Cameroon | NW | Ngoketunjia |  | Immunization | Outreach |
| SITREP NWSW | OCHA | 31st October 2019 | UNFPA | UN Agency | Cameroon | SW | Ngoketunjia |  | Reproductive Health |  |
| SITREP NWSW | OCHA | 31st October 2020 | CARITIS | Faith Based Organisation | Cameroon | NW | Ngoketunjia |  | Essential Health Services |  |
| NWSW Health Cluster Bulletin | Health Cluster | 25th June 2020 | UNICEF | UN Agency | Cameroon | NWSW |  |  | Community based COVID-19 response | Community Based Interventions |
| SITREP NWSW | OCHA | 30th April 2020 | CBCHS | Faith Based Organisation | Cameroon | NW |  |  | Essential Health Services | Community Based Interventions |
| SITREP NWSW | OCHA | 29th February 2020 | WHO | UN Agency | Cameroon | NWSW |  |  | EWARS | Community Based Interventions |
| SITREP NWSW | OCHA | 30th March 2020 | DEMTOU Humanitaire | National NGO | Cameroon | SW |  |  | Essential Health Services | Mobile Clinics |
| NWSW Health Cluster Bulletin | Health Cluster | 3rd May 2020 | NRC | INGO | Cameroon | NWSW |  |  | Health promotion |  |
| NWSW Health Cluster Bulletin | Health Cluster | 13th May 2020 | IRC | INGO | Cameroon | SW |  |  | Health promotion | Community Based Interventions |
| NWSW Health Cluster Bulletin | Health Cluster | 13th May 2020 | Value Health Africa | National NGO | Cameroon | SW |  |  | Community based COVID-19 response |  |
| NWSW Health Cluster Bulletin | Health Cluster | 8th August 2020 | Women protection Empowerment | National NGO | Cameroon | SW |  |  | Community based COVID-19 response |  |
| SITREP NWSW | OCHA | 31st May 2020 | RDPH | Ministry of Health | Cameroon | SW |  |  | Community based COVID-19 response |  |
| SITREP NWSW | OCHA | 9th December 2020 | WHO | UN Agency | Cameroon | NWSW |  |  | Mental Health |  |
| NEN Humanitarian Response Monthly Health Sector Bulletin | Health Sector Nigeria | Feb-20 | ALIMA | INGO | Nigeria | Borno |  | Askira | Essential Health Services | Fixed Facility Services |
| NEN Humanitarian Response Monthly Health Sector Bulletin | Health Sector Nigeria | May-20 | ALIMA | INGO | Nigeria | Borno |  | Askira | Essential Health Services | Fixed Facility Services |
| NEN Humanitarian Response Monthly Health Sector Bulletin | Health Sector Nigeria | Aug-19 | UNFPA | UN Agency | Nigeria | Borno |  | Askira | Reproductive Health | Fixed Facility Services |
| NEN Humanitarian Response Monthly Health Sector Bulletin | Health Sector Nigeria | 31st March 2018 | IRC | INGO | Nigeria | Yobe |  | Askira Uba | Essential Health Services | Mobile Clinics |
| NEN Humanitarian Response Monthly Health Sector Bulletin | Health Sector Nigeria | May-20 | CPPLI | National NGO | Nigeria | Borno |  | Askira Uba | Community based COVID-19 response | Community Based Interventions |
| NEN Humanitarian Response Monthly Health Sector Bulletin | Health Sector Nigeria | Oct-18 | IRC | INGO | Nigeria | Borno |  | Bama | Essential Health Services | Mobile Clinics & Fixed Facility Services |
| NEN Humanitarian Response Monthly Health Sector Bulletin | Health Sector Nigeria | May-20 | FHI360 | INGO | Nigeria | Borno |  | Bama | Essential Health Services | Fixed Facility Services |
| NEN Humanitarian Response Monthly Health Sector Bulletin | Health Sector Nigeria | May-20 | INTERSOS | INGO | Nigeria | Borno |  | Bama | Essential Health Services | Mobile Clinics |
| NEN Humanitarian Response Monthly Health Sector Bulletin | Health Sector Nigeria | Jan-19 | IOM | INGO | Nigeria | Borno |  | Bama | Mental health | Mobile Clinics |
| NEN Humanitarian Response Monthly Health Sector Bulletin | Health Sector Nigeria | Feb-20 | Care International | INGO | Nigeria | Borno |  | Bama | Essential Health Services | Mobile Clinics |
| NEN Humanitarian Response Monthly Health Sector Bulletin | Health Sector Nigeria | Feb-20 | INTERSOS | INGO | Nigeria | Borno |  | Bama | Essential Health Services | Mobile Clinics & Fixed Facility Services |
| Activity Report 2019 | Medicins Sans Frontiers | 2019 | MSF | INGO | Nigeria | Borno |  | Bama | Essential Health Services | Fixed Facility Services |
| NEN Humanitarian Response Monthly Health Sector Bulletin | Health Sector Nigeria | 31st March 2018 | INTERSOS | INGO | Nigeria | Borno |  | Bama | Essential Health Services | Fixed Facility Services |
| NEN Humanitarian Response Monthly Health Sector Bulletin | Health Sector Nigeria | May-20 | WHO | UN Agency | Nigeria | Borno |  | Bama | Mental health | Outreach |
| NEN Humanitarian Response Monthly Health Sector Bulletin | Health Sector Nigeria | Jan-19 | FHI360 | INGO | Nigeria | Borno |  | Banki | Essential Health Services | Fixed Facility Services |
| NEN Humanitarian Response Monthly Health Sector Bulletin | Health Sector Nigeria | Jan-19 | IOM | INGO | Nigeria | Borno |  | Banki | Mental health | Mobile Clinics |
| NEN Humanitarian Response Monthly Health Sector Bulletin | Health Sector Nigeria | Feb-20 | FHI360 | INGO | Nigeria | Borno |  | Banki | Essential Health Services | Fixed Facility Services |
| Activity Report 2019 | Medicins Sans Frontiers | 2019 | MSF | INGO | Nigeria | Borno |  | Banki | Essential Health Services | Fixed Facility Services |
| NEN Humanitarian Response Monthly Health Sector Bulletin | Health Sector Nigeria | Aug-19 | UNFPA | UN Agency | Nigeria | Borno |  | Banki | Reproductive Health | Fixed Facility Services |
| NEN Humanitarian Response Monthly Health Sector Bulletin | Health Sector Nigeria | Oct-18 | IRC | INGO | Nigeria | Yobe |  | Bursari | Essential Health Services | Mobile Clinics & Fixed Facility Services |
| NEN Humanitarian Response Monthly Health Sector Bulletin | Health Sector Nigeria | Jan-19 | FHI360 | INGO | Nigeria | Borno |  | Damasak | Essential Health Services | Fixed Facility Services |
| NEN Humanitarian Response Monthly Health Sector Bulletin | Health Sector Nigeria | Feb-20 | FHI360 | INGO | Nigeria | Borno |  | Damasak | Essential Health Services | Fixed Facility Services |
| NEN Humanitarian Response Monthly Health Sector Bulletin | Health Sector Nigeria | 31st March 2018 | WHO | UN Agency | Nigeria | Yobe |  | Damaturu | Immunization | Outreach |
| NEN Humanitarian Response Monthly Health Sector Bulletin | Health Sector Nigeria | Oct-18 | IRC | INGO | Nigeria | Borno |  | Damaturu | Essential Health Services | Mobile Clinics & Fixed Facility Services |
| NEN Humanitarian Response Monthly Health Sector Bulletin | Health Sector Nigeria | May-20 | WHO | UN Agency | Nigeria | Borno |  | Damboa | Mental health | Outreach |
| NEN Humanitarian Response Monthly Health Sector Bulletin | Health Sector Nigeria | Aug-19 | UNFPA | UN Agency | Nigeria | Borno |  | Damboa | Reproductive Health | Fixed Facility Services |
| NEN Humanitarian Response Monthly Health Sector Bulletin | Health Sector Nigeria | Feb-20 | FSACI | National NGO | Nigeria | Adamawa |  | Demsa | Health promotion |  |
| NEN Humanitarian Response Monthly Health Sector Bulletin | Health Sector Nigeria | Apr-20 | Action Health Incoporated | National NGO | Nigeria | Borno |  | Dikwa | Reproductive Health | Outreach |
| NEN Humanitarian Response Monthly Health Sector Bulletin | Health Sector Nigeria | May-20 | WHO | UN Agency | Nigeria | Borno |  | Dikwa | Mental health | Outreach |
| NEN Humanitarian Response Monthly Health Sector Bulletin | Health Sector Nigeria | 31st March 2018 | FHI360 | INGO | Nigeria | Borno |  | Dikwa | Essential Health Services | Fixed Facility Services |
| NEN Humanitarian Response Monthly Health Sector Bulletin | Health Sector Nigeria | May-20 | INTERSOS | INGO | Nigeria | Borno |  | Dikwa | Essential Health Services | Mobile Clinics |
| NEN Humanitarian Response Monthly Health Sector Bulletin | Health Sector Nigeria | Jan-19 | FHI360 | INGO | Nigeria | Borno |  | Dikwa | Essential Health Services | Fixed Facility Services |
| NEN Humanitarian Response Monthly Health Sector Bulletin | Health Sector Nigeria | Jan-19 | IOM | INGO | Nigeria | Borno |  | Dikwa | Mental health | Mobile Clinics |
| NEN Humanitarian Response Monthly Health Sector Bulletin | Health Sector Nigeria | Feb-20 | Care International | INGO | Nigeria | Borno |  | Dikwa | Essential Health Services | Mobile Clinics |
| NEN Humanitarian Response Monthly Health Sector Bulletin | Health Sector Nigeria | Feb-20 | INTERSOS | INGO | Nigeria | Borno |  | Dikwa | Essential Health Services | Mobile Clinics & Fixed Facility Services |
| NEN Humanitarian Response Monthly Health Sector Bulletin | Health Sector Nigeria | Feb-20 | FHI360 | INGO | Nigeria | Borno |  | Dikwa | Essential Health Services | Fixed Facility Services |
| NEN Humanitarian Response Monthly Health Sector Bulletin | Health Sector Nigeria | 31st March 2018 | INTERSOS | INGO | Nigeria | Borno |  | Dikwa | Essential Health Services | Fixed Facility Services |
| NEN Humanitarian Response Monthly Health Sector Bulletin | Health Sector Nigeria | Aug-19 | UNFPA | UN Agency | Nigeria | Borno |  | Dikwa | Reproductive Health | Fixed Facility Services |
| NEN Humanitarian Response Monthly Health Sector Bulletin | Health Sector Nigeria | Oct-18 | IRC | INGO | Nigeria | Borno |  | Fika | Essential Health Services | Mobile Clinics & Fixed Facility Services |
| NEN Humanitarian Response Monthly Health Sector Bulletin | Health Sector Nigeria | Dec-18 | NAIIS | National NGO | Nigeria | Adamawa |  | Fufore | Communty Management of HIV&TB | Community Based Interventions |
| NEN Humanitarian Response Monthly Health Sector Bulletin | Health Sector Nigeria | Oct-18 | IRC | INGO | Nigeria | Borno |  | Fune | Essential Health Services | Mobile Clinics & Fixed Facility Services |
| Activity Report 2018 | Medicins Sans Frontiers | 2018 | MSF | INGO | Nigeria | Borno |  | Gajigana | Essential Health Services | Mobile Clinics |
| Activity Report 2018 | Medicins Sans Frontiers | 2018 | MSF | INGO | Nigeria | Borno |  | Gajiram | Essential Health Services | Mobile Clinics |
| NEN Humanitarian Response Monthly Health Sector Bulletin | Health Sector Nigeria | 31st March 2018 | LESGO | National NGO | Nigeria | Adamawa |  | Girei | Communty Management of HIV&TB |  |
| NEN Humanitarian Response Monthly Health Sector Bulletin | Health Sector Nigeria | May-20 | GZDI | National NGO | Nigeria | Adamawa |  | Gombi | Communty Management of HIV&TB | Community Based Interventions |
| NEN Humanitarian Response Monthly Health Sector Bulletin | Health Sector Nigeria | May-20 | WHO | UN Agency | Nigeria | Borno |  | Gubio | Mental health | Outreach |
| NEN Humanitarian Response Monthly Health Sector Bulletin | Health Sector Nigeria | Oct-18 | IRC | INGO | Nigeria | Borno |  | Gubio | Essential Health Services | Mobile Clinics & Fixed Facility Services |
| NEN Humanitarian Response Monthly Health Sector Bulletin | Health Sector Nigeria | Oct-18 | IRC | INGO | Nigeria | Borno |  | Guidan | Essential Health Services | Mobile Clinics & Fixed Facility Services |
| NEN Humanitarian Response Monthly Health Sector Bulletin | Health Sector Nigeria | Oct-18 | IRC | INGO | Nigeria | Borno |  | Gujba | Essential Health Services | Mobile Clinics & Fixed Facility Services |
| NEN Humanitarian Response Monthly Health Sector Bulletin | Health Sector Nigeria | May-20 | Action Against Hunger | INGO | Nigeria | Yobe |  | Gujba | Reproductive Health | Fixed Facility Services |
| NEN Humanitarian Response Monthly Health Sector Bulletin | Health Sector Nigeria | Oct-18 | IRC | INGO | Nigeria | Borno |  | Guzamala | Essential Health Services | Mobile Clinics & Fixed Facility Services |
| NEN Humanitarian Response Monthly Health Sector Bulletin | Health Sector Nigeria | Jan-19 | IOM | INGO | Nigeria | Borno |  | Gwoza | Mental health | Mobile Clinics |
| Activity Report 2019 | Medicins Sans Frontiers | 2019 | MSF | INGO | Nigeria | Borno |  | Gwoza | Essential Health Services | Fixed Facility Services |
| NEN Humanitarian Response Monthly Health Sector Bulletin | Health Sector Nigeria | May-20 | ALIMA | INGO | Nigeria | Borno |  | Hawul | Essential Health Services | Fixed Facility Services |
| NEN Humanitarian Response Monthly Health Sector Bulletin | Health Sector Nigeria | Feb-20 | ALIMA | INGO | Nigeria | Borno |  | Hawul | Essential Health Services | Fixed Facility Services |
| NEN Humanitarian Response Monthly Health Sector Bulletin | Health Sector Nigeria | May-20 | GZDI | National NGO | Nigeria | Adamawa |  | Hong | Communty Management of HIV&TB | Community Based Interventions |
| NEN Humanitarian Response Monthly Health Sector Bulletin | Health Sector Nigeria | Oct-18 | IRC | INGO | Nigeria | Yobe |  | Jakusko | Essential Health Services | Mobile Clinics & Fixed Facility Services |
| NEN Humanitarian Response Monthly Health Sector Bulletin | Health Sector Nigeria | May-20 | WHO | UN Agency | Nigeria | Borno |  | Jere | Mental health | Outreach |
| NEN Humanitarian Response Monthly Health Sector Bulletin | Health Sector Nigeria | Jan-19 | Action Against Hunger | INGO | Nigeria | Borno |  | Jere | Reproductive Health | Fixed Facility Services |
| NEN Humanitarian Response Monthly Health Sector Bulletin | Health Sector Nigeria | Feb-20 | ALIMA | INGO | Nigeria | Borno |  | Jere | Essential Health Services | Fixed Facility Services |
| NEN Humanitarian Response Monthly Health Sector Bulletin | Health Sector Nigeria | Oct-18 | IRC | INGO | Nigeria | Borno |  | Jere | Essential Health Services | Mobile Clinics & Fixed Facility Services |
| NEN Humanitarian Response Monthly Health Sector Bulletin | Health Sector Nigeria | Nov-19 | Care International | INGO | Nigeria | Borno |  | Jere | Reproductive Health | Mobile Clinics & Fixed Facility Services |
| NEN Humanitarian Response Monthly Health Sector Bulletin | Health Sector Nigeria | Oct-18 | WHO | UN Agency | Nigeria | Borno |  | Jere | EWARS | Mobile Clinics |
| NEN Humanitarian Response Monthly Health Sector Bulletin | Health Sector Nigeria | May-20 | WHO | UN Agency | Nigeria | Borno |  | Kaga | Mental health | Outreach |
| NEN Humanitarian Response Monthly Health Sector Bulletin | Health Sector Nigeria | Feb-20 | Terre des Hommes | INGO | Nigeria | Borno |  | Kala/Balge | Essential Health Services | Mobile Clinics |
| NEN Humanitarian Response Monthly Health Sector Bulletin | Health Sector Nigeria | May-20 | WHO | UN Agency | Nigeria | Borno |  | Kala/Balge | Mental health | Outreach |
| NEN Humanitarian Response Monthly Health Sector Bulletin | Health Sector Nigeria | Oct-18 | IRC | INGO | Nigeria | Yobe |  | Karasuwa | Essential Health Services | Mobile Clinics & Fixed Facility Services |
| NEN Humanitarian Response Monthly Health Sector Bulletin | Health Sector Nigeria | May-20 | WHO | UN Agency | Nigeria | Borno |  | Konduga | Mental health | Outreach |
| NEN Humanitarian Response Monthly Health Sector Bulletin | Health Sector Nigeria | Oct-18 | IRC | INGO | Nigeria | Borno |  | Konduga | Essential Health Services | Mobile Clinics & Fixed Facility Services |
| NEN Humanitarian Response Monthly Health Sector Bulletin | Health Sector Nigeria | Nov-19 | Care International | INGO | Nigeria | Borno |  | Konduga | Reproductive Health | Mobile Clinics & Fixed Facility Services |
| NEN Humanitarian Response Monthly Health Sector Bulletin | Health Sector Nigeria | Oct-18 | IRC | INGO | Nigeria | Borno |  | Kukawa | Essential Health Services | Mobile Clinics & Fixed Facility Services |
| Activity Report 2018 | Medicins Sans Frontiers | 2018 | MSF | INGO | Nigeria | Borno |  | Kukawa | Essential Health Services | Mobile Clinics |
| NEN Humanitarian Response Monthly Health Sector Bulletin | Health Sector Nigeria | Dec-18 | NAIIS | National NGO | Nigeria | Adamawa |  | Lamurde | Communty Management of HIV&TB | Community Based Interventions |
| NEN Humanitarian Response Monthly Health Sector Bulletin | Health Sector Nigeria | Oct-18 | IRC | INGO | Nigeria | Yobe |  | Machina | Essential Health Services | Mobile Clinics & Fixed Facility Services |
| NEN Humanitarian Response Monthly Health Sector Bulletin | Health Sector Nigeria | May-20 | CPPLI | National NGO | Nigeria | Adamawa |  | Madagali | Community based COVID-19 response | Community Based Interventions |
| NEN Humanitarian Response Monthly Health Sector Bulletin | Health Sector Nigeria | May-20 | WHO | UN Agency | Nigeria | Borno |  | Mafa | Mental health | Outreach |
| NEN Humanitarian Response Monthly Health Sector Bulletin | Health Sector Nigeria | Feb-20 | Terre des Hommes | INGO | Nigeria | Borno |  | Mafa | Essential Health Services | Mobile Clinics |
| NEN Humanitarian Response Monthly Health Sector Bulletin | Health Sector Nigeria | Oct-18 | IRC | INGO | Nigeria | Borno |  | Mafa | Essential Health Services | Mobile Clinics & Fixed Facility Services |
| NEN Humanitarian Response Monthly Health Sector Bulletin | Health Sector Nigeria | May-20 | Action Against Hunger | INGO | Nigeria | Borno |  | Magumeri | Reproductive Health | Fixed Facility Services |
| NEN Humanitarian Response Monthly Health Sector Bulletin | Health Sector Nigeria | May-20 | INTERSOS | INGO | Nigeria | Borno |  | Magumeri | Essential Health Services | Mobile Clinics |
| NEN Humanitarian Response Monthly Health Sector Bulletin | Health Sector Nigeria | Jan-19 | Action Against Hunger | INGO | Nigeria | Borno |  | Magumeri | Reproductive Health | Fixed Facility Services |
| NEN Humanitarian Response Monthly Health Sector Bulletin | Health Sector Nigeria | Oct-18 | IRC | INGO | Nigeria | Borno |  | Magumeri | Essential Health Services | Mobile Clinics & Fixed Facility Services |
| NEN Humanitarian Response Monthly Health Sector Bulletin | Health Sector Nigeria | Oct-18 | WHO | UN Agency | Nigeria | Borno |  | Magumeri | EWARS | Mobile Clinics |
| NEN Humanitarian Response Monthly Health Sector Bulletin | Health Sector Nigeria | 31st March 2018 | UNFPA | UN Agency | Nigeria | Borno |  | Maiduguri | Reproductive Health |  |
| NEN Humanitarian Response Monthly Health Sector Bulletin | Health Sector Nigeria | May-19 | RHHF | National NGO | Nigeria | Borno |  | Maiduguri | Reproductive Health | Outreach |
| NEN Humanitarian Response Monthly Health Sector Bulletin | Health Sector Nigeria | Jan-19 | IOM | INGO | Nigeria | Borno |  | Maiduguri | Mental health | Mobile Clinics |
| NEN Humanitarian Response Monthly Health Sector Bulletin | Health Sector Nigeria | Feb-20 | ALIMA | INGO | Nigeria | Borno |  | Maiduguri | Essential Health Services | Fixed Facility Services |
| NEN Humanitarian Response Monthly Health Sector Bulletin | Health Sector Nigeria | Apr-20 | PUI | INGO | Nigeria | Borno |  | Maiduguri | Essential Health Services | Fixed Facility Services |
| Activity Report 2019 | Medicins Sans Frontiers | 2019 | MSF | INGO | Nigeria | Borno |  | Maiduguri | Essential Health Services | Fixed Facility Services |
| NEN Humanitarian Response Monthly Health Sector Bulletin | Health Sector Nigeria | May-20 | CPPLI | National NGO | Nigeria | Adamawa |  | Michika | Community based COVID-19 response | Community Based Interventions |
| NEN Humanitarian Response Monthly Health Sector Bulletin | Health Sector Nigeria | May-20 | GZDI | National NGO | Nigeria | Adamawa |  | Michika | Communty Management of HIV&TB | Community Based Interventions |
| NEN Humanitarian Response Monthly Health Sector Bulletin | Health Sector Nigeria | Jan-19 | Action Against Hunger | INGO | Nigeria | Borno |  | Maiduguri | Reproductive Health | Fixed Facility Services |
| NEN Humanitarian Response Monthly Health Sector Bulletin | Health Sector Nigeria | Oct-18 | IRC | INGO | Nigeria | Borno |  | Maiduguri | Essential Health Services | Mobile Clinics & Fixed Facility Services |
| NEN Humanitarian Response Monthly Health Sector Bulletin | Health Sector Nigeria | Nov-19 | Care International | INGO | Nigeria | Borno |  | Maiduguri | Reproductive Health | Mobile Clinics & Fixed Facility Services |
| NEN Humanitarian Response Monthly Health Sector Bulletin | Health Sector Nigeria | 31st March 2018 | PUI | INGO | Nigeria | Borno |  | Maiduguri | Essential Health Services | Mobile Clinics |
| NEN Humanitarian Response Monthly Health Sector Bulletin | Health Sector Nigeria | Oct-18 | WHO | UN Agency | Nigeria | Borno |  | Maiduguri | EWARS | Mobile Clinics |
| NEN Humanitarian Response Monthly Health Sector Bulletin | Health Sector Nigeria | May-19 | UNFPA | UN Agency | Nigeria | Borno |  | Maiduguri | Reproductive Health | Mobile Clinics |
| NEN Humanitarian Response Monthly Health Sector Bulletin | Health Sector Nigeria | May-19 | UNFPA | UN Agency | Nigeria | Borno |  | Maiduguri | Reproductive Health | Mobile Clinics |
| NEN Humanitarian Response Monthly Health Sector Bulletin | Health Sector Nigeria | Oct-18 | IRC | INGO | Nigeria | Borno |  | Mobbar | Essential Health Services | Mobile Clinics & Fixed Facility Services |
| NEN Humanitarian Response Monthly Health Sector Bulletin | Health Sector Nigeria | Nov-19 | FHI360 | INGO | Nigeria | Borno |  | Mobbar | Essential Health Services | Fixed Facility Services |
| NEN Humanitarian Response Monthly Health Sector Bulletin | Health Sector Nigeria | May-19 | RHHF | National NGO | Nigeria | Borno |  | Monguno | Reproductive Health | Mobile Clinics |
| NEN Humanitarian Response Monthly Health Sector Bulletin | Health Sector Nigeria | Jan-19 | IOM | INGO | Nigeria | Borno |  | Monguno | Mental health | Mobile Clinics |
| NEN Humanitarian Response Monthly Health Sector Bulletin | Health Sector Nigeria | May-20 | WHO | UN Agency | Nigeria | Borno |  | Monguno | Mental health | Outreach |
| NEN Humanitarian Response Monthly Health Sector Bulletin | Health Sector Nigeria | Apr-20 | Action Health Incoporated | National NGO | Nigeria | Borno |  | Monguno | Reproductive Health | Outreach |
| NEN Humanitarian Response Monthly Health Sector Bulletin | Health Sector Nigeria | Apr-20 | NCA | National NGO | Nigeria | Borno |  | Monguno | Reproductive Health |  |
| NEN Humanitarian Response Monthly Health Sector Bulletin | Health Sector Nigeria | May-20 | ALIMA | INGO | Nigeria | Borno |  | Monguno | Essential Health Services | Fixed Facility Services |
| NEN Humanitarian Response Monthly Health Sector Bulletin | Health Sector Nigeria | Jan-19 | Action Against Hunger | INGO | Nigeria | Borno |  | Monguno | Reproductive Health | Fixed Facility Services |
| NEN Humanitarian Response Monthly Health Sector Bulletin | Health Sector Nigeria | Oct-18 | IRC | INGO | Nigeria | Borno |  | Monguno | Essential Health Services | Mobile Clinics & Fixed Facility Services |
| NEN Humanitarian Response Monthly Health Sector Bulletin | Health Sector Nigeria | May-19 | UNFPA | UN Agency | Nigeria | Borno |  | Monguno | Reproductive Health | Mobile Clinics |
| NEN Humanitarian Response Monthly Health Sector Bulletin | Health Sector Nigeria | Sep-18 | Plan International | INGO | Nigeria | Adamawa |  | Mubi | Health promotion |  |
| NEN Humanitarian Response Monthly Health Sector Bulletin | Health Sector Nigeria | 31st March 2018 | WHO | UN Agency | Nigeria | Adamawa |  | Mubi North | Immunization | Mobile Clinics |
| NEN Humanitarian Response Monthly Health Sector Bulletin | Health Sector Nigeria | Feb-20 | LESGO | National NGO | Nigeria | Adamawa |  | Mubi North | Health promotion |  |
| NEN Humanitarian Response Monthly Health Sector Bulletin | Health Sector Nigeria | Aug-19 | CHEDA | National NGO | Nigeria | Adamawa |  | Mubi North | Health promotion |  |
| NEN Humanitarian Response Monthly Health Sector Bulletin | Health Sector Nigeria | Aug-19 | JHF | National NGO | Nigeria | Adamawa |  | Mubi North | Communty Management of HIV&TB |  |
| NEN Humanitarian Response Monthly Health Sector Bulletin | Health Sector Nigeria | May-19 | AGUF | National NGO | Nigeria | Adamawa |  | Mubi North | Reproductive Health | Community Based Interventions |
| NEN Humanitarian Response Monthly Health Sector Bulletin | Health Sector Nigeria | Jan-19 | IOM | INGO | Nigeria | Adamawa |  | Mubi North | Mental health | Mobile Clinics |
| NEN Humanitarian Response Monthly Health Sector Bulletin | Health Sector Nigeria | May-20 | GZDI | National NGO | Nigeria | Adamawa |  | Mubi South | Communty Management of HIV&TB | Community Based Interventions |
| NEN Humanitarian Response Monthly Health Sector Bulletin | Health Sector Nigeria | Feb-20 | LESGO | National NGO | Nigeria | Adamawa |  | Mubi South | Health promotion |  |
| NEN Humanitarian Response Monthly Health Sector Bulletin | Health Sector Nigeria | Aug-19 | CHEDA | National NGO | Nigeria | Adamawa |  | Mubi South | Health promotion |  |
| NEN Humanitarian Response Monthly Health Sector Bulletin | Health Sector Nigeria | May-19 | AGUF | National NGO | Nigeria | Adamawa |  | Mubi South | Reproductive Health | Community Based Interventions |
| NEN Humanitarian Response Monthly Health Sector Bulletin | Health Sector Nigeria | Apr-20 | JHF | National NGO | Nigeria | Adamawa |  | Mubi South | Communty Management of HIV&TB |  |
| NEN Humanitarian Response Monthly Health Sector Bulletin | Health Sector Nigeria | Oct-18 | IRC | INGO | Nigeria | Yobe |  | Nagere | Essential Health Services | Mobile Clinics & Fixed Facility Services |
| NEN Humanitarian Response Monthly Health Sector Bulletin | Health Sector Nigeria | May-20 | WHO | UN Agency | Nigeria | Borno |  | Ngala | Mental health | Outreach |
| NEN Humanitarian Response Monthly Health Sector Bulletin | Health Sector Nigeria | 31st March 2018 | INTERSOS | INGO | Nigeria | Borno |  | Ngala | Essential Health Services | Fixed Facility Services |
| NEN Humanitarian Response Monthly Health Sector Bulletin | Health Sector Nigeria | Jan-19 | INTERSOS | INGO | Nigeria | Borno |  | Ngala | Essential Health Services | Mobile Clinics |
| NEN Humanitarian Response Monthly Health Sector Bulletin | Health Sector Nigeria | Jan-19 | IOM | INGO | Nigeria | Borno |  | Ngala | Mental health | Mobile Clinics |
| NEN Humanitarian Response Monthly Health Sector Bulletin | Health Sector Nigeria | Feb-20 | FHI360 | INGO | Nigeria | Borno |  | Ngala | Essential Health Services | Fixed Facility Services |
| NEN Humanitarian Response Monthly Health Sector Bulletin | Health Sector Nigeria | May-19 | RHHF | National NGO | Nigeria | Borno |  | Ngala | Reproductive Health | Outreach |
| NEN Humanitarian Response Monthly Health Sector Bulletin | Health Sector Nigeria | May-19 | UNFPA | UN Agency | Nigeria | Borno |  | Ngala | Reproductive Health | Mobile Clinics |
| NEN Humanitarian Response Monthly Health Sector Bulletin | Health Sector Nigeria | May-20 | WHO | UN Agency | Nigeria | Borno |  | Nganzai | Mental health | Outreach |
| NEN Humanitarian Response Monthly Health Sector Bulletin | Health Sector Nigeria | Jan-19 | Action Against Hunger | INGO | Nigeria | Borno |  | Nganzai | Reproductive Health | Fixed Facility Services |
| NEN Humanitarian Response Monthly Health Sector Bulletin | Health Sector Nigeria | Oct-18 | IRC | INGO | Nigeria | Borno |  | Nganzai | Essential Health Services | Mobile Clinics & Fixed Facility Services |
| NEN Humanitarian Response Monthly Health Sector Bulletin | Health Sector Nigeria | Oct-18 | IRC | INGO | Nigeria | Borno |  | Nguru | Essential Health Services | Mobile Clinics & Fixed Facility Services |
| NEN Humanitarian Response Monthly Health Sector Bulletin | Health Sector Nigeria | Oct-18 | IRC | INGO | Nigeria | Borno |  | Nguru | Essential Health Services | Mobile Clinics & Fixed Facility Services |
| NEN Humanitarian Response Monthly Health Sector Bulletin | Health Sector Nigeria | 31st March 2018 | DOBIYAN | National NGO | Nigeria | Adamawa |  | Numan | Communty Management of HIV&TB |  |
| NEN Humanitarian Response Monthly Health Sector Bulletin | Health Sector Nigeria | Dec-18 | NAIIS | National NGO | Nigeria | Adamawa |  | Numan | Communty Management of HIV&TB | Community Based Interventions |
| NEN Humanitarian Response Monthly Health Sector Bulletin | Health Sector Nigeria | Oct-18 | IRC | INGO | Nigeria | Yobe |  | Potiskum | Essential Health Services | Mobile Clinics & Fixed Facility Services |
| NEN Humanitarian Response Monthly Health Sector Bulletin | Health Sector Nigeria | Apr-20 | NCA | National NGO | Nigeria | Borno |  | Pulka | Reproductive Health |  |
| NEN Humanitarian Response Monthly Health Sector Bulletin | Health Sector Nigeria | Jan-19 | IOM | INGO | Nigeria | Borno |  | Pulka | Mental health | Mobile Clinics |
| Activity Report 2019 | Medicins Sans Frontiers | 2019 | MSF | INGO | Nigeria | Borno |  | Pulka | Essential Health Services | Fixed Facility Services |
| Activity Report 2018 | Medicins Sans Frontiers | 2018 | MSF | INGO | Nigeria | Borno |  | Rann | Essential Health Services | Fixed Facility Services |
| NEN Humanitarian Response Monthly Health Sector Bulletin | Health Sector Nigeria | Oct-18 | IRC | INGO | Nigeria | Yobe |  | Tarmuwa | Essential Health Services | Mobile Clinics & Fixed Facility Services |
| NEN Humanitarian Response Monthly Health Sector Bulletin | Health Sector Nigeria | Aug-19 | UNFPA | UN Agency | Nigeria | Borno |  | Uba | Reproductive Health | Fixed Facility Services |
| NEN Humanitarian Response Monthly Health Sector Bulletin | Health Sector Nigeria | Feb-20 | LESGO | National NGO | Nigeria | Adamawa |  | Yola North | Health promotion |  |
| NEN Humanitarian Response Monthly Health Sector Bulletin | Health Sector Nigeria | Dec-18 | NAIIS | National NGO | Nigeria | Adamawa |  | Yola North | Communty Management of HIV&TB | Community Based Interventions |
| NEN Humanitarian Response Monthly Health Sector Bulletin | Health Sector Nigeria | Apr-20 | JHF | National NGO | Nigeria | Adamawa |  | Yola North | Communty Management of HIV&TB |  |
| NEN Humanitarian Response Monthly Health Sector Bulletin | Health Sector Nigeria | 31st March 2018 | AGUF | National NGO | Nigeria | Adamawa |  | Yola South | Health promotion |  |
| NEN Humanitarian Response Monthly Health Sector Bulletin | Health Sector Nigeria | Feb-20 | LESGO | National NGO | Nigeria | Adamawa |  | Yola South | Health promotion |  |
| NEN Humanitarian Response Monthly Health Sector Bulletin | Health Sector Nigeria | Feb-20 | SWOGE | National NGO | Nigeria | Adamawa |  | Yola South | Health promotion |  |
| NEN Humanitarian Response Monthly Health Sector Bulletin | Health Sector Nigeria | Aug-19 | CHEDA | National NGO | Nigeria | Adamawa |  | Yola South | Health promotion |  |
| NEN Humanitarian Response Monthly Health Sector Bulletin | Health Sector Nigeria | Dec-18 | NAIIS | National NGO | Nigeria | Adamawa |  | Yola South | Communty Management of HIV&TB | Community Based Interventions |
| NEN Humanitarian Response Monthly Health Sector Bulletin | Health Sector Nigeria | Apr-20 | JHF | National NGO | Nigeria | Adamawa |  | Yola South | Communty Management of HIV&TB |  |
| NEN Humanitarian Response Monthly Health Sector Bulletin | Health Sector Nigeria | Oct-18 | IRC | INGO | Nigeria | Borno |  | Yunusari | Essential Health Services | Mobile Clinics & Fixed Facility Services |
| NEN Humanitarian Response Monthly Health Sector Bulletin | Health Sector Nigeria | Oct-18 | IRC | INGO | Nigeria | Yobe |  | Yusufari | Essential Health Services | Mobile Clinics & Fixed Facility Services |
| NEN Humanitarian Response Monthly Health Sector Bulletin | Health Sector Nigeria | 31st March 2018 | UNFPA | UN Agency | Nigeria | Adamawa |  |  | Reproductive Health |  |
| NEN Humanitarian Response Monthly Health Sector Bulletin | Health Sector Nigeria | 31st March 2018 | UNFPA | UN Agency | Nigeria | Yobe |  |  | Reproductive Health |  |
| NEN Humanitarian Response Monthly Health Sector Bulletin | Health Sector Nigeria | 31st March 2018 | UNICEF | UN Agency | Nigeria | Borno |  |  | Essential Health Services | Fixed Facility Services |
| NEN Humanitarian Response Monthly Health Sector Bulletin | Health Sector Nigeria | 31st March 2018 | UNICEF | UN Agency | Nigeria | Yobe |  |  | Essential Health Services |  |
| NEN Humanitarian Response Monthly Health Sector Bulletin | Health Sector Nigeria | 31st March 2018 | WHO | UN Agency | Nigeria | Yobe |  |  | Reproductive Health | Mobile Clinics |
| NEN Humanitarian Response Monthly Health Sector Bulletin | Health Sector Nigeria | May-20 | UNICEF | UN Agency | Nigeria | Adamawa |  |  | Essential Health Services | Fixed Facility Services |
| NEN Humanitarian Response Monthly Health Sector Bulletin | Health Sector Nigeria | Feb-20 | UNICEF | UN Agency | Nigeria | Yobe |  |  | Essential Health Services | Fixed Facility Services |
| NEN Humanitarian Response Monthly Health Sector Bulletin | Health Sector Nigeria | Feb-20 | UNICEF | UN Agency | Nigeria | Borno |  |  | Essential Health Services | Fixed Facility Services |
| NEN Humanitarian Response Monthly Health Sector Bulletin | Health Sector Nigeria | Feb-20 | WHO | UN Agency | Nigeria | Borno |  |  | Essential Health Services |  |
| NEN Humanitarian Response Monthly Health Sector Bulletin | Health Sector Nigeria | Oct-18 | WHO | UN Agency | Nigeria | Borno |  |  | Essential Health Services | Community Based Interventions |
| NEN Humanitarian Response Monthly Health Sector Bulletin | Health Sector Nigeria | Oct-18 | WHO | UN Agency | Nigeria | Adamawa |  |  | Essential Health Services | Community Based Interventions |
| NEN Humanitarian Response Monthly Health Sector Bulletin | Health Sector Nigeria | Oct-18 | WHO | UN Agency | Nigeria | Yobe |  |  | Essential Health Services | Community Based Interventions |
| NEN Humanitarian Response Monthly Health Sector Bulletin | Health Sector Nigeria | Jan-19 | IRC | INGO | Nigeria | Adamawa |  |  | Essential Health Services | Mobile Clinics |
| NEN Humanitarian Response Monthly Health Sector Bulletin | Health Sector Nigeria | Jan-19 | IRC | INGO | Nigeria | Borno |  |  | Essential Health Services | Mobile Clinics |
| NEN Humanitarian Response Monthly Health Sector Bulletin | Health Sector Nigeria | Jan-19 | IOM | INGO | Nigeria | Yobe |  |  | Mental health | Mobile Clinics |
| Activity Report 2018 | Medicins Sans Frontiers | 2018 | MSF | INGO | Nigeria | Yobe |  |  | Emergency Care | Fixed Facility Services |
| NEN Humanitarian Response Monthly Health Sector Bulletin | Health Sector Nigeria | Jan-19 | MdM | INGO | Nigeria |  |  |  | Essential Health Services |  |
