# Supplementary material for: Exploring factors influencing the selection of primary health care delivery models in conflict-affected settings of North West and South West regions of Cameroon and North-East Nigeria: A study protocol
Source: PLoS One. 2023 May 3;18(5):e0284957. doi: 10.1371/journal.pone.0284957 (PMC10155952; doi:10.1371/journal.pone.0284957)
Supplement: S2 Appendix — (DOCX) [file pone.0284957.s002.docx]

**APPENDIX 2**

**Methodology used in mapping the Primary Health Care services**

For the Cameroon literature, several publications provided information on PHC location by division only while other publication gave the location by districts only number. When location was limited by district only, the corresponding division of the district was obtained by a google search of the division in which the district was located. But the reverse could not be true when only divisions where mentioned in publications. The same logic was applied with publications from Nigeria where corresponding states for local government areas were obtained using google. Faced with this situation, a decision was made to map services in Cameroon by Divisions and in Nigeria by local government area. To produce visual maps for this study, the excel data spreadsheet was summarised to suit the records and fields needed to develop maps. These were convert to a “comma separated values” file which is compatible with the Quantum GIS (QGIS) software was used in producing the maps. The administrative boundaries of the countries involved were downloaded onto admin layer level two (AdminLayer2). These layers were loaded into the QGIS and overlaid on each other following the admin layer hierarchy. The CSV file was also loaded into the application and join on the respective layers with the “join function” in the QGIS. The maps were then plotted using “Layout Manager” which allows the users to drop points for the different PHC services and model of care. ‘Graduated symbology’ was used to detail the partner presence in different region and local government area on the map.
