# Supplementary material for: Exploring factors influencing the selection of primary health care delivery models in conflict-affected settings of North West and South West regions of Cameroon and North-East Nigeria: A study protocol
Source: PLoS One. 2023 May 3;18(5):e0284957. doi: 10.1371/journal.pone.0284957 (PMC10155952; doi:10.1371/journal.pone.0284957)
Supplement: S3 Appendix — (DOCX) [file pone.0284957.s003.docx]

## **APPENDIX 3**

## **Screener for In-depth interviews (IDIs) and focus group discussions (FGDs)**

**Recruiter:** Introduce yourself, the research group you represent.

Thank you very much for assisting us with this important project. Please read the following notes before beginning your recruiting.

***Research Objectives:*** This research is aimed at exploring how primary health care (PHC) are designed by humanitarian organizations and through stakeholder engagement, design a toolkit for evaluating quality in PHC delivery across different models of care.

**The Research:** this research will be requesting participants to take part in an in-depth interview (IDI) or focus group discussion (FGD). Open completion of these IDIs and FGDs, a workshop will be organised where a few participants from the IDIs and FGDs will be organised to share research findings. IDIs and FGDs will take place at an agreed location with participants.

Name of participant __________________________

Phone___________________________

Email (optional)__________________________

Organisation/ institution (for Humanitation Organisations) ________________________

Community (for IDPs and Host community members) _____________________________

Country _________________________________

Region/state ______________________________

Recruiter _____________________________ Date ______________________

1. **Screener for IDI with Humanitarian organisations (phase 2 of study)**
2. What is your role in this organisation?

General coordinator

Grants writer

Program director/manager

Field supervisor

Field staff DISMISS

1. For how long have you worked with this organisation?

1-5 months DISMISS

6 – 12 months

12 months and above

1. Are you directly involved in program design and proposal development in the organisation?

Yes

No DISMISS

1. **Screener for FGDs with internally displaced persons (phase 2 of study)**
2. Are you are internally displaced person or a resident of a community that hosts IDPs?

Yes

No DISMISS

1. Have you or any member of your family ever benefitted or witness from health care provided by any humanitarian organisation?

Yes

No DISMISS

1. **Screener for IDI with Humanitarian organisations (phase 3 of study)**
2. What is your role in this organisation?

General coordinator

Grants writer

Program director/manager

Field supervisor

Field staff

1. Have you ever served as a staff in mobile clinics/mobile health team/mobile outreach team, or fixed facility care or as a CHWs?

Yes

No DISMISS

**NOTE TO** **RECRUITER:** use this questions to identify especially articulate respondents that are easy to understand. Answers must be Interesting, thoughtful and well-expressed. It is extremely Important that respondents in this study are articulate. If respondent does not give a two or three sentence, unprompted answer, you must terminate. Please be certain the respondent talks for 2-3 sentences without you asking any additional questions.
