# Supplementary material for: Exploring factors influencing the selection of primary health care delivery models in conflict-affected settings of North West and South West regions of Cameroon and North-East Nigeria: A study protocol
Source: PLoS One. 2023 May 3;18(5):e0284957. doi: 10.1371/journal.pone.0284957 (PMC10155952; doi:10.1371/journal.pone.0284957)
Supplement: S4 Appendix — (DOCX) [file pone.0284957.s004.docx]

**APPENDIX 4**

**Anticipated number of study participants by study phase and component**

| **Study Phase** | **Data collection method** | **No of anticipated participants** |
| --- | --- | --- |
| ***Mapping different primary health care models*** | Surveys using structured questionnaires with humanitarian organizations | Cameroon   - 40 organizations and Ministry of Health in South West and North West Regions   North East Nigeria   - 60 organizations in North East Nigeria   (Note: the intention is to have all organizations interviewed. So the number of organization intervening at the time the survey will be conducted may vary from what is anticipated. Surveys will be done one per organization) |
| ***Exploring the factors influencing the selection of PHC models of care*** | in-depth interviews (IDI) with program staff of humanitarian organizations | Cameroon   - 13 IDIs in South West and North West   one person interviewed per organisation  Nigeria   - 17 IDIs North East Nigeria   Note:   - one person interviewed per organisation - these numbers are indicative. Interviews will be stop only when saturation is achieved |
|  | focus groups discussions (FGD) with internally displaced persons (IDPs) and members of host communities | Cameroon   - 04 FGDs with IDPs and host community members in South West and North West   8-12 participants in each FGD  Nigeria   - 06 FGDs with IDPs and host community members in Borno, Yobe and Adamawa   (NOTE:   - the intention is to mix the IDPs by sex and gender – if feedback from community leaders indicates this would be problematic, the FGDs will be done with separate groups on sex.) - these numbers are indicative. Interviews will be stop only when saturation is achieved |
| ***Determining the coverage and gaps in services*** | In-depth interviews (IDIs) with humanitarian program staff | Cameroon   - 05 IDIs with humanitarian staff in South west and North West regions   Nigeria   - 05 IDIs with humanitarian staff in North East Nigeria   Note: these numbers are indicative. Interviews will be stop only when saturation is achieved |
|  | In-depth interviews (IDIs) with IDPs | Cameroon  01 IDI with IDP in South West or North West regions  Nigeria  01 IDI with IDP from North East Nigeria  Note: these numbers are indicative. Interviews will be stop only when saturation is achieved |
| Development of framework | Stakeholders workshop with Ministry of Health, humanitarian organizations, IDPs | Cameroon  30 key stakeholders  Nigeria  30 key stakeholders |
